# Effect of Biltong Dried Beef Processing on the Reduction of *Listeria monocytogenes*, *E. coli* O157:H7, and *Staphylococcus aureus*, and the Contribution of the Major Marinade Components

**DOI:** 10.3390/microorganisms10071308

**Published:** 2022-06-28

**Authors:** Kavya Gavai, Caitlin Karolenko, Peter M. Muriana

**Affiliations:** 1 Robert M. Kerr Food & Agricultural Products Center, Oklahoma State University, Stillwater, OK 74078, USA; kavya.gavai@okstate.edu (K.G.); caitlin.e.karolenko@okstate.edu (C.K.); 2 Department of Animal and Food Sciences, Oklahoma State University, Stillwater, OK 74078, USA

**Keywords:** *Listeria monocytogenes*, *E. coli* O157:H7, *Staphylococcus aureus*, biltong, log reduction, acid adaptation, water activity

## Abstract

Biltong is a dry beef product that is manufactured without a heat lethality step, raising concerns of whether effective microbial pathogen reduction can occur during biltong processing. Raw beef inoculated with 4-strain cocktails of either *E. coli* O157:H7, *Listeria monocytogenes*, or *Staphylococcus aureus*, and processed with a standard biltong process, were shown to incur a >5-log reduction in 6–8 days after marination by vacuum-tumbling for 30 min in vinegar, salt, spices (coriander, pepper) when dried at 23.9 °C (75 °F) at 55% relative humidity (RH). Pathogenic challenge strains were acid-adapted in media containing 1% glucose to ensure that the process was sufficiently robust to inhibit acid tolerant strains. Internal water activity (A_w_) reached < 0.85 at 5-log reduction levels, ensuring that conditions were lower than that which would support bacterial growth, or toxin production by *S. aureus* should it be internalized during vacuum tumbling. This was further confirmed by ELISA testing for staphylococcal enterotoxins A and B (SEA, SEB) after marination and again after 10 days of drying whereby levels were lower than initial post-marination levels. Comparison of log reduction curves obtained for *E. coli* O157:H7, *L. monocytogenes*, *S. aureus*, and *Salmonella* (prior study) showed that microbial reduction was not significantly different (*p* < 0.05) demonstrating that even without a heat lethality step, the biltong process we examined produces a safe beef product according to USDA-FSIS guidelines.

## 1. Introduction

Dried beef products are found worldwide, and although they may have originated as a way to preserve perishable meat products [1], they have now become accepted as nutritious high protein ‘meat snacks’ for sportsmen, campers, and hikers [2]. They are also included in specialized ‘paleolithic diets’ to simulate foods our hunter-gatherer ancestors consumed, serving as a modern means of reducing weight [3]. Popular dried beef products such as beef jerky, kippered beef, and biltong can be found in nearly every supermarket and convenience store. A recent newcomer to the manufacturing of biltong, Stryve Foods (Plano, TX, USA), recently announced record annual sales of $30 million USD (2021), representing an increase of 77% over 2020 and projected sales of $43–48 million USD for 2022. It is just one of many biltong beef processors experiencing increased sales of this new product line in US markets that have been dominated by traditional American style beef jerky.

Although biltong is noted to have originated in South Africa as the result of early Dutch settlers attempting to preserve meat, there are no South African regulatory guidelines for its manufacture. As biltong has grown in popularity in the UK and US, efforts to export the product directly from South Africa have been thwarted. In prior years, it was difficult to export biltong out of South Africa into developed countries where food safety is heavily regulated if there is no acceptable regulatory guidance for its manufacture, unless it was made to EU standards or in a HACCP-approved facility. Kussaga et al. (2014) have suggested that the absence of risk-based food safety management programs such as HACCP or ISO22000 from government through retail/company level has prevented many exports from South Africa at a time when their products are in demand [4]. As is often the case, guidance from the US, New Zealand, or other developed countries on the manufacture of RTE meats such as beef jerky was often cited in making biltong [5]. Similarly, questions posed to British Columbia health authorities (Canada) regarding biltong manufacture relied on information from the Canadian Food Inspection Service (CFIS) which adopted guidance from the United States Department of Agriculture, Food Safety and Inspection Service (USDA-FSIS) [6].

In the US, the manufacture of dried beef products such as beef jerky is regulated by USDA-FSIS and provides industry guidance to ensure product safety. Since biltong is not defined by a standard of identity, USDA-FSIS often cites ‘Compliance Guidelines’ for conditions of manufacture of beef jerky that includes heating in the presence of 90% RH to govern issues of concern with biltong manufacture [7,8]. Beef jerky is often processed with a high temperature lethality step of >62.8 °C (145 °F) dependent on % RH, whereas biltong is air-dried at room temperature at 21.1–26.7 °C (70–80 °F) after marination with salt, spices, and vinegar [9,10]. Since high temperatures are not used in biltong manufacture, USDA-FSIS has allowed modifications to biltong processing similar to what was allowed for pepperoni and summer sausage at the height of shigatoxigenic *E. coli* outbreaks in the 1990’s with such products [11] and currently allows two options to accommodate biltong processing. One option is to test all lots of ingredients prior to use for presence/absence of *Salmonella* spp. as a pathogen of concern, and then use a manufacturing process that demonstrates ≥ 2-log reduction of *Salmonella* (i.e., ‘a pathogen of concern’). The ingredients should test negative; if they test positive, those ingredients cannot be used unless rendered free of *Salmonella* spp. The second option is to use a process that provides ≥5-log reduction of *Salmonella* spp. with no need for *Salmonella* testing of ingredients. This later alternative is preferred because it eliminates costly *Salmonella* testing and problems that arise when *Salmonella*-positive ingredients are encountered. However, the difficulty is in demonstrating that the process achieves ≥5-log reduction of the pathogen of concern.

The absence of a heat lethality (cook) step in the processing of biltong has been a concern for pathogens associated with raw beef and/or the beef processing environment. Studies have demonstrated the presence of *Staphylococcus* and *Bacillus* (*Bacillus cereus*) as the predominant taxa associated with biltong obtained from local butcheries in South Africa [12]. Similarly, another study examining 150 samples of biltong purchased in South Africa demonstrated the presence of *Escherichia coli*, coagulase-positive *Staphylococcus*, *Salmonella* and *Listeria monocytogenes* [13]. A lack of information on details of the impact of biltong marinades, vinegar, and drying on pathogen reduction given the moderate processing conditions has caused USDA-FSIS to recognize that there are ‘knowledge gaps’ with such new products that are being introduced in the US [14]. These knowledge gap areas are often noted as being insufficiently covered by scientific information and they are often targeted as viable research topic areas by USDA or industry-funded research programs.

The USDA-FSIS requires that manufacturers of shelf stable dried beef products validate the microbial safety of their process of manufacture. *Salmonella* spp. has been noted as a ‘pathogen of concern’ because *Salmonella*, and other pathogens, have historically been associated with food production from animals and the meat derived from them [15,16,17,18]. The USDA-FSIS requires process validation with only a single ‘pathogen of concern’ for commercial processors to manufacture and sell biltong. However, corporate management of retail supermarket chains often require additional food safety assurances that other pathogens also associated with beef (*L. monocytogenes, E. coli* O157:H7, and *S. aureus*) are also effectively inhibited by the manufacturing process [13,19,20]. Currently, there is limited data (‘data gaps’) regarding biltong processes that demonstrate sufficient reduction of these foodborne pathogens.

In this study, we examined a common biltong process for process lethality against acid-adapted cultures of *E. coli* O157:H7, *L. monocytogenes*, and *S. aureus*, the effect of individual ingredient components (salt, spice, vinegar) on process lethality against *E. coli* O157:H7 and *L. monocytogenes*, and whether biltong processing allows for *S. aureus* enterotoxin production from *S. aureus* inoculated beef. This work, together with our previous work with *Salmonella* spp., should satisfy process safety concerns regarding the four major pathogens associated with dried beef products and help fill scientific data gaps that exist for biltong-processed dried meat products.

## 2. Materials and Methods

### 2.1. Bacterial Strains, Growth Conditions, and Antibiotic Resistance

Bacterial cultures used in this study included 4 strains each of *L. monocytogenes*, *E. coli* O157:H7, and *S. aureus* (Table 1). Strains of *L. monocytogenes* included ATCC 49594/Scott A-2 (serotype 4b, human isolate), V7-2 (serotype 1/2a, milk isolate), 39-2 (retail hotdog isolate), and 383-2 (ground beef isolate) [21]. These strains were resistant to streptomycin (100 ug/mL; Sigma-Aldrich, St. Louis, MO, USA) and rifamycin S/V (10 µg/mL; Sigma-Aldrich) and were plated on tryptic soy agar (TSA; Difco Brand, Becton-Dickenson, Sparks, MD, USA) containing these antibiotics for enumeration of inoculated cultures recovered from biltong beef. Strains of *E. coli* O157:H7 included ATCC 35150, ATCC 43894, ATCC 43889, and ATCC 45756 that are known for acid tolerance [22,23]. These strains were all resistant to 5 µg/mL novobiocin and 2.5 µg/mL rifamycin S/V (Sigma-Aldrich) and enumeration of these strains was conducted on TSA (Difco) containing these antibiotics. Strains of *S. aureus* and the staphylococcal enterotoxins they produce included ATCC 8095 (SEA), ATCC 13565 (SEA), ATCC 14458 (SEB), and ATCC 51740 (SEB) [24,25]. These strains were resistant to clindamycin (5 µg/mL; Sigma-Aldrich) and were plated on TSA (Difco) containing this antibiotic.

The various pathogens were confirmed for typical phenotypes on selective and differential agars: *L. monocytogenes* on modified Oxford agar (MOX, Difco, BD), *E. coli* O157:H7 on CHROMagar O157 (DRG International, Springfield, NJ, USA) and RAPID *E. coli* O157:H7 medium (Bio-Rad Laboratories, Hercules, CA, USA), and *S. aureus* on Mannitol Salt Agar (Difco-BBL, BD Laboratories, Franklin Lakes, NJ, USA); data not shown. The antibiotic resistances of the strains used in this study were also confirmed by plating on TSA, with and without antibiotics, to ensure equivalent enumeration so that survivors from processed biltong could be recovered on antibiotic-containing TSA.

Bacterial cultures were grown in tryptic soy broth (TSB, BD Bacto Brand BD211825, Franklin Lakes, NJ, USA) in 9 mL tubes at 30 °C (*L. monocytogenes*) or 37 °C (*E. coli*, *S. aureus*). Cultures were maintained for storage by centrifugation (6000× *g*, 5 °C) of 9 mL of fresh, overnight cultures and cell pellets were resuspended in 2–3 mL of fresh sterile TSB containing 10% glycerol. Cell suspensions were placed into glass vials and stored in an ultra-low freezer (−80 °C). Frozen stocks were revived by transferring 100 µL of the thawed cell suspension into 9 mL of TSB, incubating overnight at 30 °C or 37 °C, and were sub-cultured twice before use. Microbial enumeration was carried out on tryptic soy agar (TSA, BD Bacto; 1.5% agar) and plated in duplicate.

Bacterial cultures used for inoculation of biltong beef were ‘acid adapted’ by growing them in media augmented with glucose prior to use according to Wilde et al. [26] and Karolenko et al. [27]. Individual bacterial cultures were first propagated overnight at 37 °C (*E. coli*, *Staphylococcus*) or 30 °C (*Listeria*) in 9 mL TSB (BD Bacto BD286220). These cultures were individually used to re-inoculate 250 mL centrifuge bottles containing 200 mL pre-warmed TSB containing 1% glucose (BD Bacto BD286220 + 1% glucose) which were again incubated overnight (at 37 °C or 30 °C) for approximately 18 h. Individual cultures in 250 mL bottles were harvested by centrifugation, resuspended with 5 mL of sterile 0.1% buffered peptone water (BPW, BD Difco), mixed in equal proportions, and held refrigerated (5 °C) until use (within 1–2 hr).

### 2.2. Beef Handling, Fabrication, and Inoculation

**Figure 1:**
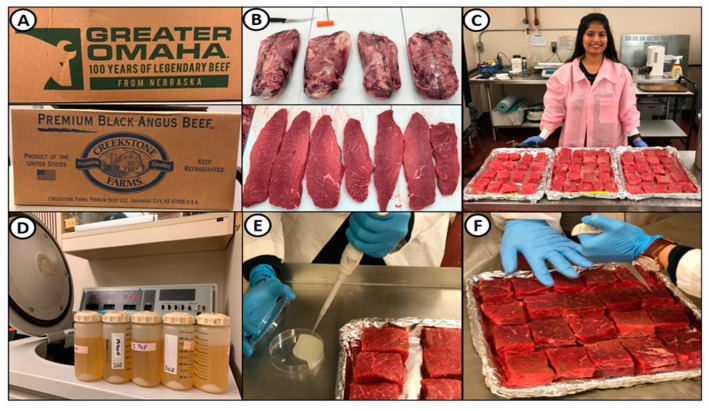
Biltong processing 1: (**A**) boxes of bottom rounds from beef processors; (**B**) bottom rounds in vacuum packaging and sliced after trimming; (**C**) further cutting of sliced biltong beef into small biltong beef pieces; (**D**) recovery and concentration of acid-adapted inoculum strains; (**E**) pipetting inoculum onto beef pieces; (**F**) spreading inoculum on beef surface using a ‘gloved finger’.

Boneless beef bottom rounds (outside round, flat; select grade or ungraded) were purchased from a local meat processor (Ralph’s, Perkins, OK, USA) who procured them from a broker (Figure 1A). Beef was stored in commercial coolers at the R.M. Kerr Food and Ag Products Center meat pilot plant until needed (i.e., used within 1–2 weeks). Beef was initially trimmed of excess fat, sliced into strips of ~1.9 cm thick, and then cut into small pieces that were ~1.9 cm thick, ~5.1 cm wide, and ~7.6 cm long (~80–100 g) (Figure 1B,C). After bottom rounds were trimmed and cut, the beef was placed on trays, wrapped in plastic bags, and maintained at 4 °C until processed (i.e., next morning).

Inoculation of mixed strains of cultures (i.e., preparation was described earlier; Figure 1D) was performed as follows: (a) the appropriate amount of beef pieces for a particular trial were placed on foil-lined trays and held in the refrigerator until inoculation; (b) ~150 μL of the resuspended/concentrated inoculum mixture was applied by micropipette onto the surface of each beef slice; (c) the inoculum was rubbed over the surface with a double-gloved finger by another person who was assisting; (d) the pieces were then turned over and the process repeated on the other side; (e) the tray(s) of inoculated beef were then placed in a refrigerator 5 °C for at least 30 min to allow attachment (Figure 1E,F).

### 2.3. Marination of Inoculated Beef Pieces

**Figure 2:**
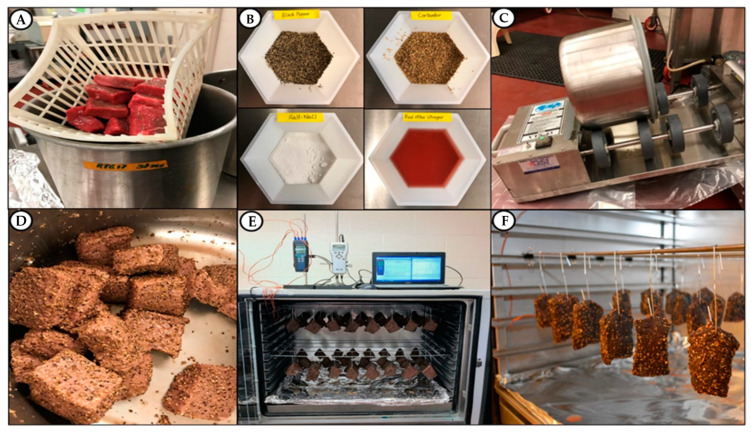
Biltong processing 2: (**A**) dip treatment of inoculated pieces in water (or antimicrobial); (**B**) black pepper, coriander, salt, and vinegar for marinade; (**C**) vacuum tumbling of biltong beef in marinade; (**D**) spiced biltong beef after marination; (**E**) humidity oven with hanging biltong beef and handheld temperature and humidity monitors; (**F**) biltong beef on lower level; the one on the far left has temperature probe inserted.

Inoculated beef pieces that were held at refrigeration for attachment were dipped in sterile water for 30 sec to mimic processes where beef is rinsed or dipped in antimicrobial solution (Figure 2A). A basic marinade mixture was taken as an average of approximately 10 biltong recipes found on the internet and total formulation was comprised of beef (92%), vinegar (4%), salt (2.1%), coriander (1.1%), and black pepper (0.8%) (Figure 2B). Since each non-beef component was based on the beef weight, for each trial we obtained the weight of the total inoculated beef pieces to determine the amount of spices and vinegar to add. These were then mixed with a whisk in a pre-chilled tumbling chamber and the inoculated beef pieces were then added. The tumbler chamber cover was sealed and a vacuum of 15 in Hg was established with a small vacuum pump and the tumbler was set to rotate for 30 min using a Biro VTS-43 tumbler (Biro, Marblehead, OH, USA; Figure 2C,D).

Additional trials were performed to evaluate the contribution of each of the individual components of the marinade mixture on the various pathogens. In these trials, pathogen-inoculated beef was subjected to marination using no marinade components, salt alone, spice alone, vinegar alone, or the complete marinade. In the non-marinade control, salt-only, and spice-only marinades, water volume was added to the equivalent formulation volume of vinegar in the vinegar-alone or complete marinade versions to achieve the same level of liquid absorption on beef during vacuum tumbling prior to drying that may affect water activity during drying.

### 2.4. Drying of Marinaded Beef Pieces

Marinaded beef pieces were then hung in a humidity oven (Hotpack Model #435315, SP Industries, Warminster, PA, USA) set to 23.9 °C (75 °F) and 55% RH, using paper clips to hang beef pieces from bars set across the top and middle of the humidity chamber. Thermocouple temperature probes (×4) connected to a handheld temperature recorder were run into the chamber; 2 were used to record chamber temperature and 2 were inserted into 2 beef pieces to record internal beef temperature. An additional handheld humidity monitor was used to record both chamber humidity and temperature as well (Figure 2E,F).

### 2.5. Beef Sampling, Microbial Enumeration, Water Activity Testing, and Enterotoxin Detection

Each series of pathogen-inoculated trials was performed in duplicate trials with beef obtained from different animals. Within each trial, beef samples were tested in triplicate at all sampling points within each trial (initial inoculation, post-marinade, and after 2, 4, 6, 8, and 10 days of drying; *n* = 6). Each series of pathogen-inoculated trials included a set of uninoculated beef that was also subjected to biltong processing (marination, tumbling, and up to 10 days of drying). The uninoculated beef was used to insert temperature probes and for water activity (A_w_) measurements throughout the drying process to ensure critical factors of temperature, humidity, and internal A_w_ were achieved and without fear of handling pathogen-inoculated pieces. Water activity was determined by slicing beef pieces in half and placing the innermost meat portion towards the humidity sensor in the sample chamber of the model HC2-AW-USB probe and analyzed using HW4-P-Quick software (Rotronic, Hauppauge, NY, USA). This provided Aw measurements for the inside of the thick beef pieces. The USDA-FSIS has indicated that vacuum-tumbled beef is considered ‘non-intact beef’ and internal A_w_ is a subject of concern for possible enterotoxin production by internalized *S. aureus* that might occur during vacuum-tumbling.

Acid-adapted cultures were used to desensitize the cultures to subsequent acidic treatment during processing and plated on TSA containing antibiotics instead of enumerating on selective/differential agars which are often known to be inhibitory to stressed cells [26]. Samples of beef retrieved from the humidity oven were placed in 6 × 9-inch Whirl-pak filter-stomacher bags (Nasco, Fort Atkinson, WI) to which 100 mL of neutralizing buffered peptone water (nBPW) was added. The stomacher bag was then stomached on a paddle masticator at high power for 2 min and subsequent dilutions were then made in 0.1% BPW. Select dilutions were then plated in duplicate on TSA + antibiotics for the respective pathogens described earlier and as previously used with Salmonella [10,26]. Plates were then incubated at 30 °C (*L. monocytogenes*) or 37 °C (*E. coli*, *S. aureus*) for 48 hrs before enumeration. At late stages of drying (i.e., ≥6 days), samples were often enumerated by plating 0.2 mL on each of 5 plates (i.e., 1 mL) to increase the sensitivity of detection at lower microbial levels.

Beef samples inoculated with a 4-strain mixture of *S. aureus* were sampled for enterotoxin detection. ELISA kits used for detecting SEA and SEB present in food, intestinal fluids, and liquid samples were obtained from Chondrex, Inc. (Woodinville, WA, USA). Test samples were analyzed according to the manufacture’s protocol for *S aureus* strains producing SEA and SEB enterotoxin which included *S. aureus* ATCC 8095 (SEA), ATCC 13565 (SEA), ATCC 14458 (SEB), and ATCC 51740 (SEB). Testing included samples from each of 2 trials of biltong processing using the complete marinade: four samples tested for each duplicate trial replication at time points (a) after marination and (b) after drying for 10 days in the temperature-controlled humidity oven. Beef samples were extracted by addition of 100 mL of sterile water to filter-membrane bags and stomaching at high speed for 2 min in a paddle mixer as described earlier; recovered liquid samples were then centrifuged to remove debris and tested for enterotoxin levels. SEA and SEB enterotoxin standards (supplied with the kit) were prepared in a range of 0.16–10 ng/mL. The standards, samples, and detection antibody were diluted in sample/standard/detection antibody dilution buffer. The assay included addition of 50 μL of diluted standards, samples, and detection antibody to microplate wells precoated with primary antibody. The plates were then incubated for 1 hr on a plate shaker at room temperature (20 °C). After incubation, 100 μL of TMB colorimetric substrate (tetramethylbenzidine) was added to each well followed by an additional incubation for 25 min at room temperature on the plate shaker. After incubation the plates were washed 3 times using 1× wash buffer supplied with the kit. The reaction was then stopped by adding 50 μL of stop solution containing 2N sulfuric acid to each well. Plates were read at 450 nm using a GENios microplate reader (Tecan Inc, Morrisville, NC, USA) and analyzed with its associated Magellan software (ver. 7.1, Tecan).

### 2.6. Statistical Analysis

Each trial in this study was performed in duplicate replication with 3 samples tested per sampling period within each trial (*n* = 6) in accordance with validation testing criteria established by the NACMCF [28] and accepted by USDA-FSIS [29]. All replications were performed as autonomous and separate experiments using separately inoculated cultures and meat from different animals. Data are presented as the mean of multiple replications with standard deviation of the mean represented by error bars. Statistical analysis was performed using the statistics functions in Sigma-Plot ver. 13 (Systat Software, San Jose, CA, USA). Timed data series were statistically analyzed by repeated measures one-way analysis of variance (RM-ANOVA) using the Holm–Sidak test for pairwise multiple comparisons to determine significant differences (*p* < 0.05) between treatments. Data treatments with different letters are significantly different (*p* < 0.05); treatments with the same letter are not significantly different (*p* > 0.05).

## 3. Results and Discussion

### 3.1. Acid Adaptation of Bacterial Cultures

**Figure 3:**
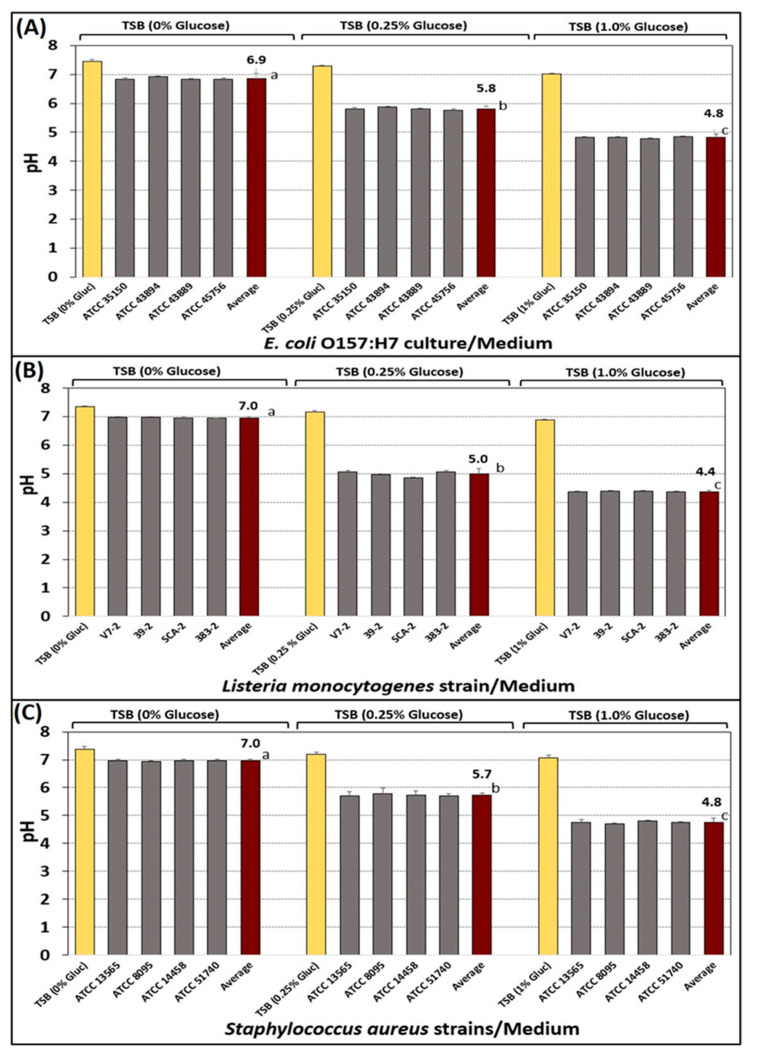
Response of various strains of *E. coli* O157:H7, *L. monocytogenes*, and *S. aureus* when grown in TSB medium at various glucose concentration (0%, 0.25%, 1.0%) at 37 °C (*S. aureus*, *E. coli* O157:H7) or 30 oC (*L. monocytogenes*): (**A**) *E. coli* O157:H7 (ATCC 35150, 41894, 43889, 45756); (**B**) *L. monocytogenes* (V7-2, 39-2, SCA-2, 383-2); (**C**) *S. aureus* (ATCC 8095, 13565, 14458, 51740). The values of the average pH for each set of 4 strains is listed above the average graph bar. Data are presented as the mean of triplicate replications and error bars represent the standard deviation from the mean. Means (for average pH) with different letters are significantly different as determined by one-way ANOVA using the Holm–Sidak test for pairwise multiple comparisons to determine significant differences (*p* < 0.05); means with different letters are significantly different (*p* < 0.05).

The bacterial cultures used in our study (*E. coli* O157:H7, *L. monocytogenes*, *S. aureus*) were cultured in TSB with varying concentrations of glucose (0%, 0.25%, 1.0%) to confirm their response to glucose concentration. As observed previously with *Salmonella* serovars [27], all three sets of strains produced correspondingly lower pH levels when incubated in TSB of increasing glucose concentration (Figure 3). For the purpose of ensuring that the biltong process is sufficiently robust to achieve high log reductions of even acid-adapted cultures, all strains used for biltong trials were grown in TSB containing 1% glucose.

Acid adaptation with *E. coli* O157:H7, *L. monocytogenes*, and *S. aureus* shows that they can lower the pH of the growth medium from approximately pH 7.0 (spent medium made without glucose) to as low as pH 4.4–4.8 in medium supplemented with 1% glucose (Figure 3). Acid adaptation was originally noted in *Salmonella* whereby growth in high glucose resulted in *Salmonella* being more tolerant of subsequent acidic conditions and has been shown to occur without genetic expression or protein synthesis whereby cells can modulate internal pH to a degree using membrane proton pumps. Other studies have noted a different process requiring genetic expression and protein synthesis, resulting in the Acid Tolerance Response (ATR) which is also ‘adaptive’ in the sense that genetic expression is often the result of responding to environmental stress conditions. These phenomena have been observed in *Salmonella*, shigatoxigenic *E. coli*, *L. monocytogenes*, and other pathogens [30,31]. Regardless of the exact mechanism, microbial acid adaption/tolerance/resistance is a concern for food processing that often rely on acidification via fermentation, food product formulation, or acidic antimicrobial interventions for food safety. The National Advisory Committee for the Microbiological Criteria for Foods (NACMCF) has recommended acid adaptation of cultures that are to be used in food challenge trials to ensure that food processes can inhibit even acid-tolerant organisms, and is preferred by US regulatory agencies (USDA-FSIS, FDA) for culture treatment when used in such studies. Bacterial cells accustomed to lower pH from their growth conditions should not be as sensitive to low pH conditions (acid treatment) and should therefore require a more robust process to result in significant microbial reductions. Investigators have shown that significant differences occur when comparisons are made between acid adapted and non-adapted cultures whereby non-adapted cultures decline faster/more when treated with an acidic treatment [30,31,32]. However, this issue is not clear since some investigators have demonstrated the opposite reaction (i.e., acid-adapted cultures showing greater reductions during acidic treatment). One explanation might be that these investigators have used selective/differential media (XLD/XLT4 for *Salmonella*, PALCAM/MOX for *L. monocytogenes*, SMAC for STEC *E. coli*) for enumeration of these pathogens; these media are harsh, and can inhibit stressed cells as would occur during acid-adaptation [33,34]. The enumeration of stressed cells showing significantly lower counts on selective/differential media has long been noted, and was recently confirmed in our comparison of enumeration of *Salmonella* serovars on TSA vs. HE vs. XLD after 4 different stresses [27].

### 3.2. Biltong Process Reduction and Contribution of the Individual Components of the Marinade

**Figure 4:**
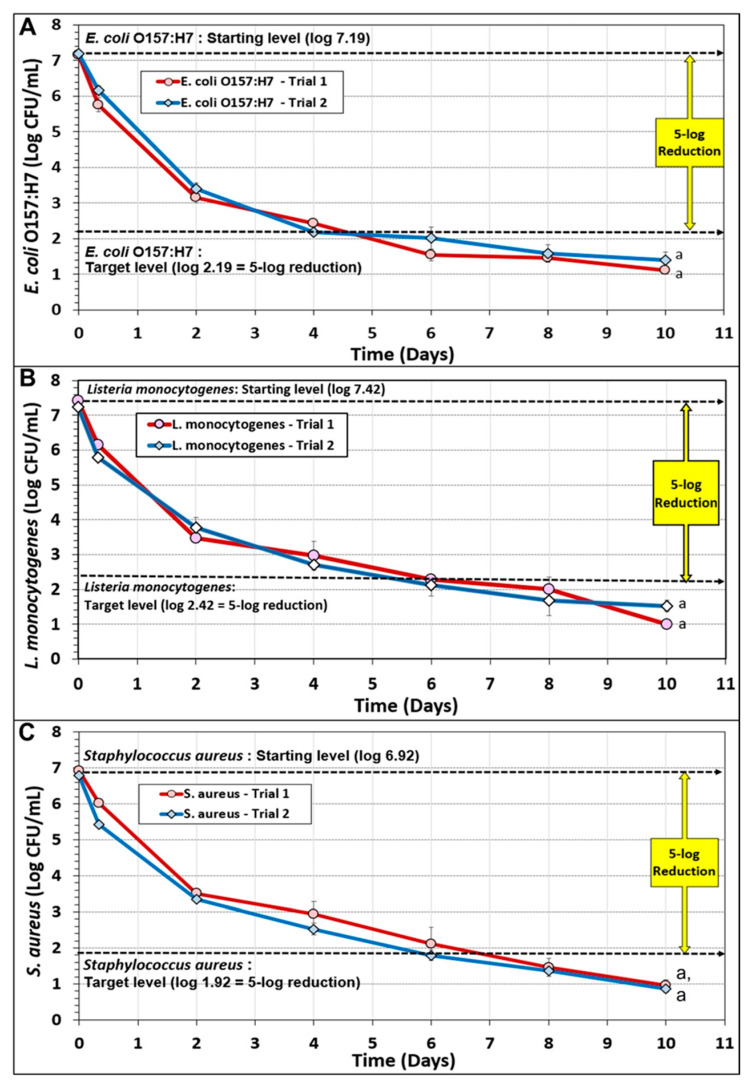
Biltong process reduction of beef inoculated with acid-adapted multi-strain cocktails of (**A**) *E. coli* O157:H7, (**B**) *L. monocytogenes*, and (**C**) *S. aureus*. The data represents 2 separate trials performed with triplicate sampling at each sampling point (*n* = 6). Duplicate trials with each pathogenic inoculum were analyzed by repeated measures one-way analysis of variance (RM-ANOVA) using the Holm–Sidak test for pairwise multiple comparisons to determine significant differences (*p* < 0.05) of treatment; treatments with the same letters are not significantly different (*p* > 0.05).

Biltong inoculated with multi-strain acid-adapted cultures of *E. coli* O157:H7, *L. monocytogenes*, or *S. aureus* were subjected to a standardized biltong process described earlier (Figure 4). Data was obtained from 2 trials performed for each pathogen whereby triplicate samples were taken at each sampling point for each trial (post-inoculation; post-marinade, i.e., 0-day drying), and after 2, 4, 6, 8, and 10 days of drying at 23.9 °C (75 °F) and 55% RH. The data shows that a ≥5-log reduction was obtained for each pathogen, but not at the same time frame of processing. The 5-log reduction target was obtained for *E. coli* O157:H7 in 5 days (extrapolated), for *L. monocytogenes* in 6 days, and *S. aureus* in 7 days (extrapolated) (Figure 4).

Another reason we have readily achieved the 5-log reduction target is that we inoculated samples with a fixed volume of inoculum giving a fixed population/sample (instead of dipping into inoculum) and we recover by stomaching samples in a fixed volume of diluent (100 mL) regardless of the dryness of the underlying beef. Other studies have enumerated on a cfu/gram basis whereby the remaining inoculum is concentrated as the underlying beef dries [20,35,36,37]. Our method eliminates this discrepancy since the underlying beef may lose up to 65% of its weight by 8–10 days of drying due to moisture loss [10].

**Figure 5:**
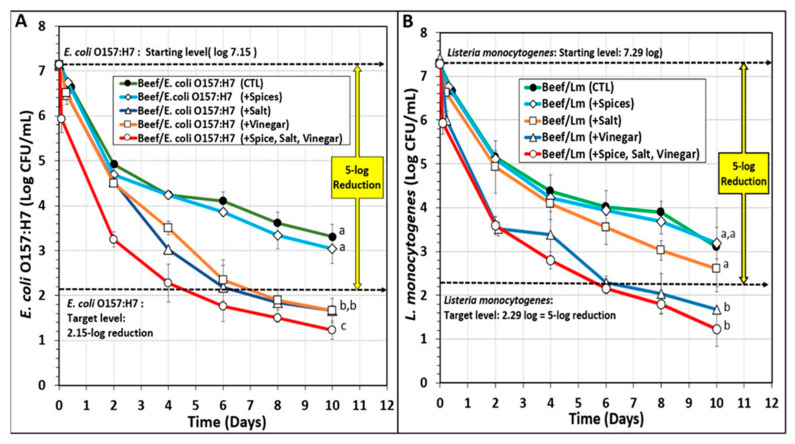
Comparison of individual marinade components and complete marinade on pathogen lethality during biltong processing of beef inoculated with (**A**) *E. coli* O157:H7 or (**B**) *L. monocytogenes*. The data represents an average of 2 separate trials performed with triplicate sampling at each sampling point; combined, *n* = 6 for each sampling period. Treatments with a particular pathogenic inoculum were analyzed by repeated measures one-way analysis of variance (RM-ANOVA) using the Holm–Sidak test for pairwise multiple comparisons to determine significant differences (*p* < 0.05) of treatment; treatments with the same letters are not significantly different (*p* > 0.05).

**Figure 6:**
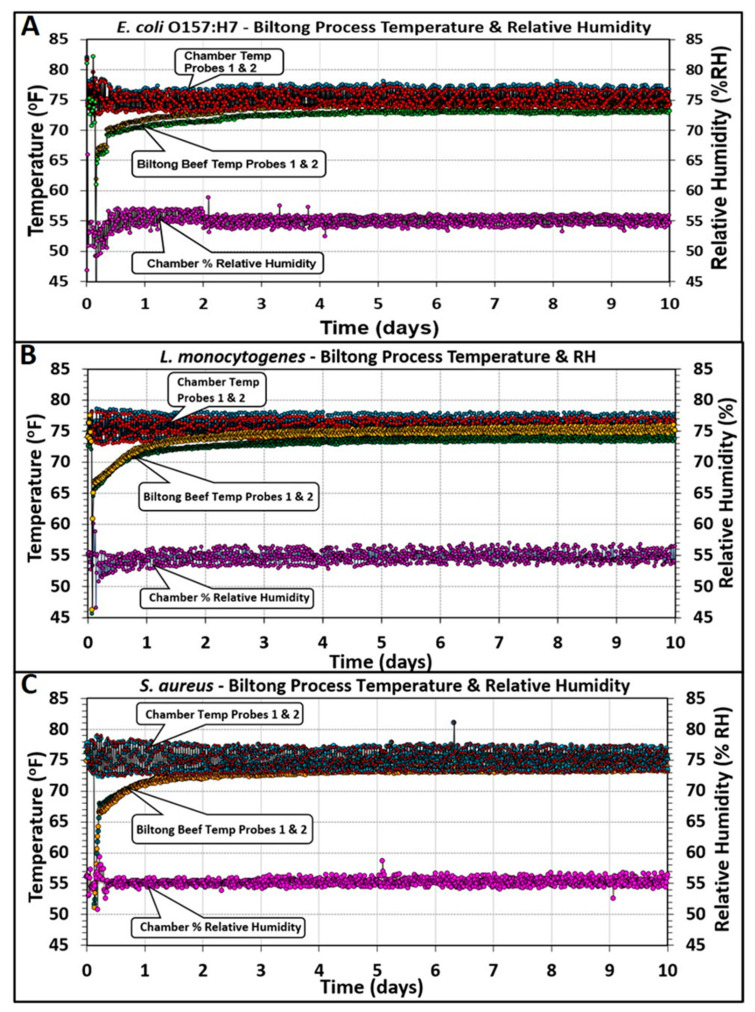
Temperature (°F) and relative humidity (% RH) oven conditions with (**A**) *E. coli* O157:H7, (**B**) *L. monocytogenes*, and (**C**) *S. aureus* inoculated beef during biltong processing. The temperature and humidity range observed were 75 ± 2.5 °F (23.9 ± 1.4 °C) and 55% ± 1.5% RH, respectively.

The contribution of the individual components of biltong marinade mixtures to process lethality was examined during additional biltong trials. These additional trials included *E. coli* O157:H7-inoculated and *L. monocytogenes*-inoculated beef that were ‘marinaded’ with water-only, salt-only, and spice-only marinades (all in water), to equate the wetting of beef with vinegar during the complete or vinegar-only marination (Figure 5). The data shows that pathogen-inoculated beef (i.e., control/CTL) vacuum-tumbled with just added water, resulted in approximately 3.5–4.0 log reduction after 10 days of drying (Figure 5A and Figure 6B). Pathogen-inoculated beef that was vacuum-tumbled with spices (in water) gave slightly higher levels of reduction, but not significantly different (*p* > 0.05) from controls (Figure 5). With salt (in water) and vinegar, some differences were observed depending on the pathogen. For instance, salt and vinegar showed similar processing effects during drying with *E. coli* O157:H7-inoculated beef, significantly different (*p* < 0.05) from both controls/water trials as well as the full complement of marinade components, but not significantly different (*p* > 0.05) from each other and both also demonstrated ≥5-log reduction of *E. coli* O157:H7 within 7 days (Figure 5A). Similar trials with salt-alone (in water) vs. vinegar-alone marination showed different results with *L. monocytogenes*. With *L. monocytogenes*-inoculated beef, salt (in water) marination gave a slightly greater reduction (but not significantly different) than for control and spice marination, yet vinegar marination gave even greater log reduction that was not significantly different from the full complement of marinade (Figure 5B). The control (CTL) data demonstrates that even without antimicrobial interventions that might be provided by marinade components, significant pathogen reduction (i.e., 3.5–4.0 log) still occurs during the drying process. This provides validation of >2-log reduction for those processes not demonstrating >5-log reduction of pathogen as one of the two alternative processes allowed for biltong processing by USDA-FSIS.

### 3.3. Biltong Process Temperature, Relative Humidity, and Water Activity (Aw) Measurements

Oven temperature measurements (2 probes) showed a range of approximately 23.9 ± 1.4 °C (75 ± 2.5 °F) for the 3 different pathogen trials and internal beef temperatures (2 probes) that gradually reached the temperature range of the oven (Figure 6). The RH probe was positioned near the midpoint of the oven near the far oven wall directly across from the wall that housed the oven fan (Figure 6). Based on the traces observed, slight tweaks were periodically made to the temperature and humidity controls during the drying period; however, these subtle tweaks could only be made after a period of observed traces since at any given time the temperature or humidity could be at a low or high point in the observed periodicity cycle.

**Figure 7:**
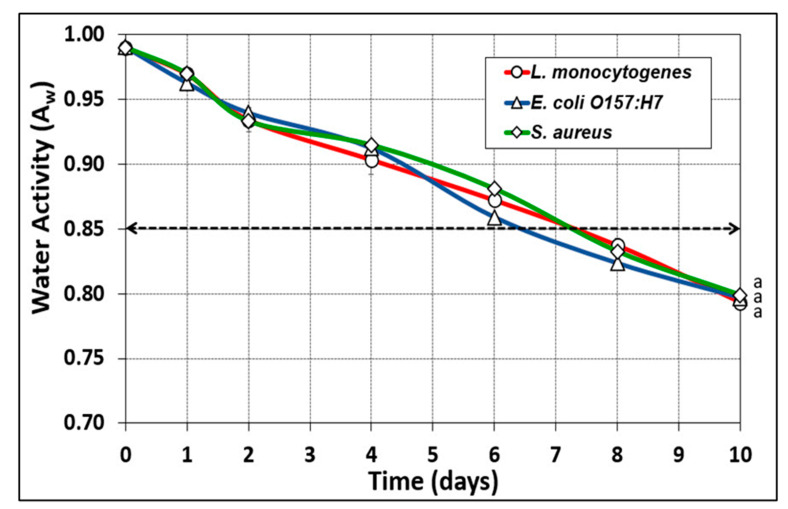
Water activity (A_w_) from interior portions of biltong beef during trials with *E. coli* O157:H7, *L. monocytogenes*, or *S. aureus,* held at 23.9 °C (75 °F) and 55% RH for up to 10 days. Repeated measures one-way analysis of variance (RM-ANOVA) using the Holm––Sidak test for pairwise multiple comparisons to determine significant differences (*p* < 0.05); treatments with the same letter are not significantly different (*p* > 0.05).

Uninoculated beef was also processed during each of the pathogenic trials and used for A_w_ and temperature measurements so that handling of pathogen-inoculated beef would not be necessary for these measurements. Water activity measurements were closely aligned during all 3 pathogen trials whereby the treatments showed no significant differences by RM-ANOVA analyses (Figure 7).

### 3.4. Analysis of Staphylococcal Enterotoxin A (SEA) and B (SEB) during Biltong Processing with S. aureus-Inoculated Beef

**Figure 8:**
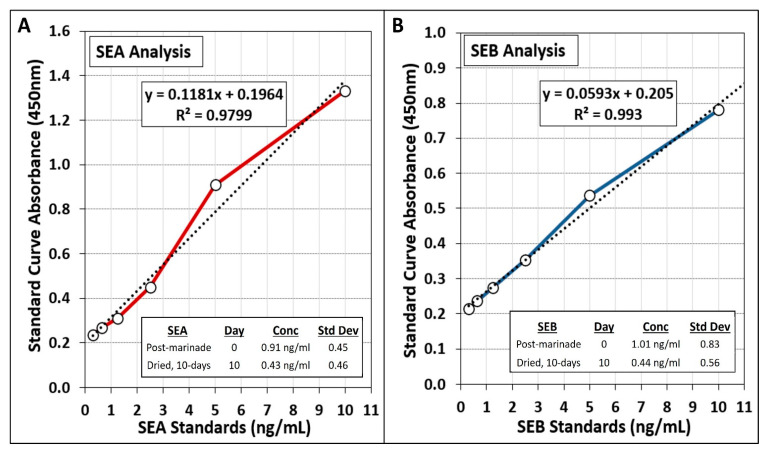
Analysis of (**A**) SEA and (**B**) SEB from *S. aureus*-inoculated biltong beef after rinse/marinade treatment (Day 0, initial measurement) and after 10 days of drying (latter measurement) using staphylococcal enterotoxin ELISA quantification kit. Graphs show standard curve plot and SEA and SEB analyses of inoculated samples post-marinade and post-dried (10 day) samples. Beef sample enterotoxin data levels were derived from assay absorbance and quantified from the equation obtained for the standard curve trend line (*n* = 4).

Strains of *S. aureus* used for beef inoculation included 2 strains that were known to be SEA-positive (ATCC 8095, ATCC 13565) and 2 known to be SEB-positive (ATCC 14458, ATCC 51740). Preliminary trials with the ELISA enterotoxin assay kit confirmed enterotoxin production by these strains (data not shown). Although our inoculum cells were centrifuged and resuspended, SEA and SEB were still detected from raw/inoculated post-marinaded beef indicative of residual carryover of SEA (0.91 ng/mL) and SEB (1.01 ng/mL) from the inoculum cell suspension. Perhaps additional washing of the cell pellets that were resuspended for use as biltong inoculum would have resulted in lower residual/initial levels on the inoculated beef. Subsequent samples obtained from biltong dried for 10 days demonstrated lower levels of SEA (0.43 ng/mL) and SEB (0.44 ng/mL) detected on beef after 10 days of drying (Figure 8). Both post-marinade and 10-day dried beef were subject to sample recovery in the same volume of extraction diluent and not on a per gram basis that would otherwise cause quantification analyses to be affected by degree of moisture loss (similar to how our microbial enumerations were performed). The data demonstrates that SEA and SEB detected after 10 days of biltong process drying were 53% and 57% lower than the levels detected at Day 0 (post-inoculation, rinsing, and marination) suggesting that enterotoxin production did not occur during our biltong process.

Because of the thickness of biltong beef, vacuum tumbling raises a concern as ‘non-intact beef’ (a safety concern for USDA-FSIS) that could have issues related to internalization of *S. aureus* that might result in production of staphylococcal enterotoxin if growth and conditions allowed. Our prior work showed that biltong made with 2.2% NaCl in the total biltong marinade formulation (including the weight of beef) approaches a moisture loss of ~60%, an internal A_w_ of 0.82 (A_w_ of 0.69 for ground biltong), a biltong pH of ~5.26, and 3–4% NaCl in the resulting biltong after 8 days of drying [9,10]. These conditions individually are not conducive to staphylococcal enterotoxin production, and less so when they occur simultaneously [29,30,31,32]. This level of water activity, though safe for enterotoxin production, may still allow mold growth which is aesthetically unappealing and reflects negatively on the product and company. Processors can do various things to mitigate mold growth such as the use of ‘single pack’ servings that are expected to be consumed once opened, the addition of moisture/oxygen absorbing packets, and/or the use of mold inhibitors in the marinade formulation.

### 3.5. Comparison of Microbial Reduction of E. coli O157:H7, L. monocytogenes, S. aureus, and Salmonella serovars during Biltong Processing

**Figure 9:**
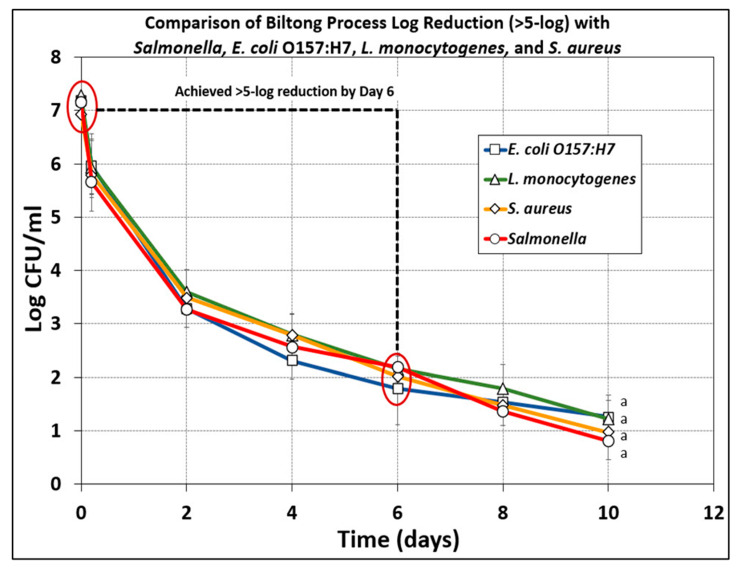
Comparison microbial lethality of a basic biltong process for >5-log reduction of *Salmonella* (adapted from [10]), *E. coli* O157:H7, *L. monocytogenes*, and *S. aureus* (this study). The data represent the average of 2 separate trials, each performed with triplicate sampling at each sampling point (combined, *n* = 6 for each sampling period). Treatments were analyzed by repeated measures one-way analysis of variance (RM-ANOVA) using the Holm–Sidak test for pairwise multiple comparisons to determine significant differences (*p* < 0.05) of treatment; treatments with the same letters are not significantly different (*p* > 0.05).

The data presented herein demonstrate that a basic biltong process and marinade composed of spices (pepper, coriander), salt, and vinegar, applied during vacuum tumbling for 30 min and followed by drying at 23.9 °C (75 °F) and 55% RH can result in ≥5-log reduction (in 5–7 days) with *Salmonella* serovars [9,10], as well as *E. coli* O157:H7, *L. monocytogenes*, and *S. aureus* (this study) (Figure 9). The 4 major groups of foodborne pathogens that could be associated with raw beef and potentially a concern for survival during the biltong process react similarly when subjected to the basic biltong process used in this study (Figure 9).

## 4. Conclusions

The data provided in our study show that a basic biltong process can achieve ≥5-log reduction and equivalent lethality among four major groups of foodborne pathogens associated with raw beef to provide a safe dried beef protein snack food. We feel the data presented herein should provide sufficient validation for USDA-FSIS process approval and product acceptance by food safety managers of retail supermarket chains, given their inter in ensuring safe foods for consumers.

## Figures and Tables

**Table 1:** List of strains used as challenge organisms for biltong processing in this study.

Organism	Strain Designation	Culture Collection Designation	Source
*L. monocytogenes*	ATCC 49594	PMM 264	ScottA-2; Clinical isolate
*L. monocytogenes*	V7-2	PMM 266	Clinical isolate
*L. monocytogenes*	39-2	PMM 39	Retail hotdogs
*L. monocytogenes*	383-2	PMM 383	Retail ground beef
*E. coli* O157:H7	ATCC 35150	PMM 407	Human feces
*E. coli* O157:H7	ATCC 43889	PMM 1111	Human feces
*E. coli* O157:H7	ATCC 43894	PMM 405	Human feces
*E. coli* O157:H7	ATCC 45756	PMM 715	JB Luchansky, USDA-ARS
*S. aureus*	ATCC 8095	PMM 323	Cream pie
*S. aureus*	ATCC 13565	PMM 318	Ham, enterotoxin illness
*S. aureus*	ATCC 14458	PMM 319	Human feces, diarrhea
*S. aureus*	ATCC 51740	PMM 678	Margarine

## Data Availability

Not applicable.
